# The essential role of TAp73 in bortezomib-induced apoptosis in p53-deficient colorectal cancer cells

**DOI:** 10.1038/s41598-017-05813-z

**Published:** 2017-07-14

**Authors:** Yasamin Dabiri, Sara Kalman, Clara-Marie Gürth, Jee Young Kim, Viola Mayer, Xinlai Cheng

**Affiliations:** Institute of Pharmacy and Molecular Biotechnology, Pharmaceutical Biology, Heidelberg University, Im Neuenheimer Feld 364, D-69120 Heidelberg, Germany

## Abstract

Mutations in the tumor suppressor p53 are among the most highly occurring events in colorectal cancer (CRC). Such mutations have been shown to influence the sensitivity of cancer cells to chemotherapeutic agents. However their impact on the efficacy of the proteasomal inhibitor bortezomib remains controversial. We thus re-evaluated the toxicity of bortezomib in the CRC cell lines HCT116 wt (wild-type) and its p53−/− clone. Transient resistance to bortezomib treatment was observed in p53-null cells that was later accompanied by an increase in levels and nuclear translocation of TAp73, an isoform of the p53-homologue p73, as well as induction of apoptosis. Knockdown of p73 in p53−/− cells using CRISPR/Cas9 significantly prolonged the duration of resistance. Moreover, similar results were observed in HT-29 cells carrying mutated p53, but not human fibroblasts with expression of functional p53. Thus, our results clearly demonstrated that TAp73 served as a substitute for p53 in bortezomib-induced apoptosis in p53-deficient or mutated cells, implicating that TAp73 could be a potential therapeutic target for treatment of CRCs, in particular those lacking functional p53.

## Introduction

Human colorectal cancer (CRC) is one of the most common cancers worldwide, with 1.2 million new cases annually diagnosed^1^. CRC often starts from premalignant lesions in the intestinal epithelium, that acquire mutations in tumor suppressor genes, including APC, SMAD4 and TP53, which consequently lead to malignant transformation^2, 3^. In spite of recent considerable advances in understanding of the molecular basis of CRC, metastatic and recurrent CRCs are still largely incurable^4^.

Among the highly mutated genes in CRC is TP53, the guardian of the genome, that regulates many vital cellular processes, including DNA repair, apoptosis, cell cycle arrest and metabolism^5^. Expression of p53 is tightly controlled through the formation of complexes with the E3 ligases MDM2 and MDM4 and consequent degradation in a ubiquitin-proteasome dependent manner^6^. Missense mutations in the TP53 gene lead to either loss of anti-tumor or gain of novel oncogenic activity, which is associated with both drug resistance and tumor exacerbation^7, 8^. Genetic analysis of p53 mutations revealed that the GC- > AT transition of CpG dinucleotides at codons 175, 248 and 273^9^ and deletion induced by hemizygous loss at the 17p chromosomal region are two frequent types of mutations. Thus, a tremendous effort has been put to restore the wild-type function of p53.

The transcription factor p73 belongs to the p53 family of proteins and exists in at least 14 different isoforms, arising from two independent promoters on the TP73 gene and further alternative splicing of the transcripts^10^. The transactivation (TA) domain containing TAp73 and the amino-terminal domain-deleted ΔNp73 represent two major isoforms. The overall biological outcome of the p73 protein seems to be highly dependent to the relative expression of these two isoforms with TAp73 being pro-apoptotic and ΔNp73 being a potential oncogene that counteracts the tumor suppressor activity of both TAp73 and p53^10–12^.

On the other hand, bortezomib, also known as Velcade or PS-341, is a bronic dipeptide proteasome inhibitor, and the first of its class to receive FDA approval for the treatment of multiple myeloma. The drug has also shown potent inhibition of tumor cell growth and progression at IC50 values down to the nanomolar range in a wide spectrum of malignancy models including breast, prostate, lung and liver cancer, as well as CRC^13–16^. Clinically, with regards to multiple myeloma, the drug demonstrated remarkable efficacy and relatively few side effects^17, 18^, however resistance emerges in the majority of patients receiving it^17^. The most well characterized mechanism of bortezomib-induced cell death is the inhibition of the proteolytic activity of the 26S proteasome, which comprises two outer 19S regulatory complexes and one inner 20S core particle^13, 14^. The role of p53 in proteasome inhibitor-mediated apoptosis is controversial. Studies have shown that p53 is required for inducing apoptosis in LNCaP^18^, KIM-2^19^, TT^20^ and FRO cells^20^ in response to proteasome inhibition, but not in HeLa^21^, DHL^22^ and PC-3 cells^23^. Therefore, the precise molecular mechanism of bortezomib appears to be cancer type-dependent.

Although previous results showed potent anti-proliferative effects of bortezomib in HCT116 cells, the impact of p53 on these effects is still controversial^24–28^. In our initial experiment, we carefully re-evaluated bortezomib’s anti-proliferative activity in HCT116 wt (wild-type) and p53−/− cells under different experimental conditions. We observed transient resistance in p53−/− cells to bortezomib after 24 hrs of treatment, which was diminished upon long-term treatments. Studying the molecular mechanism revealed the essential role of TAp73, a transcriptionally active isoform of the p53-homologue, p73, in inducing apoptosis in p53-deficient cells, but not in wt. Knocking down p73 by a CRISPR/Cas9 plasmid in HCT116 p53−/− cells or a p73 siRNA in HT-29 carrying mutated p53 significantly enhanced the resistance to bortezomib, confirming the anti-tumorigenic role of TAp73 in cells lacking functional p53.

## Results

### Transient resistance to bortezomib in HCT116 p53−/− cells

**Figure 1:**
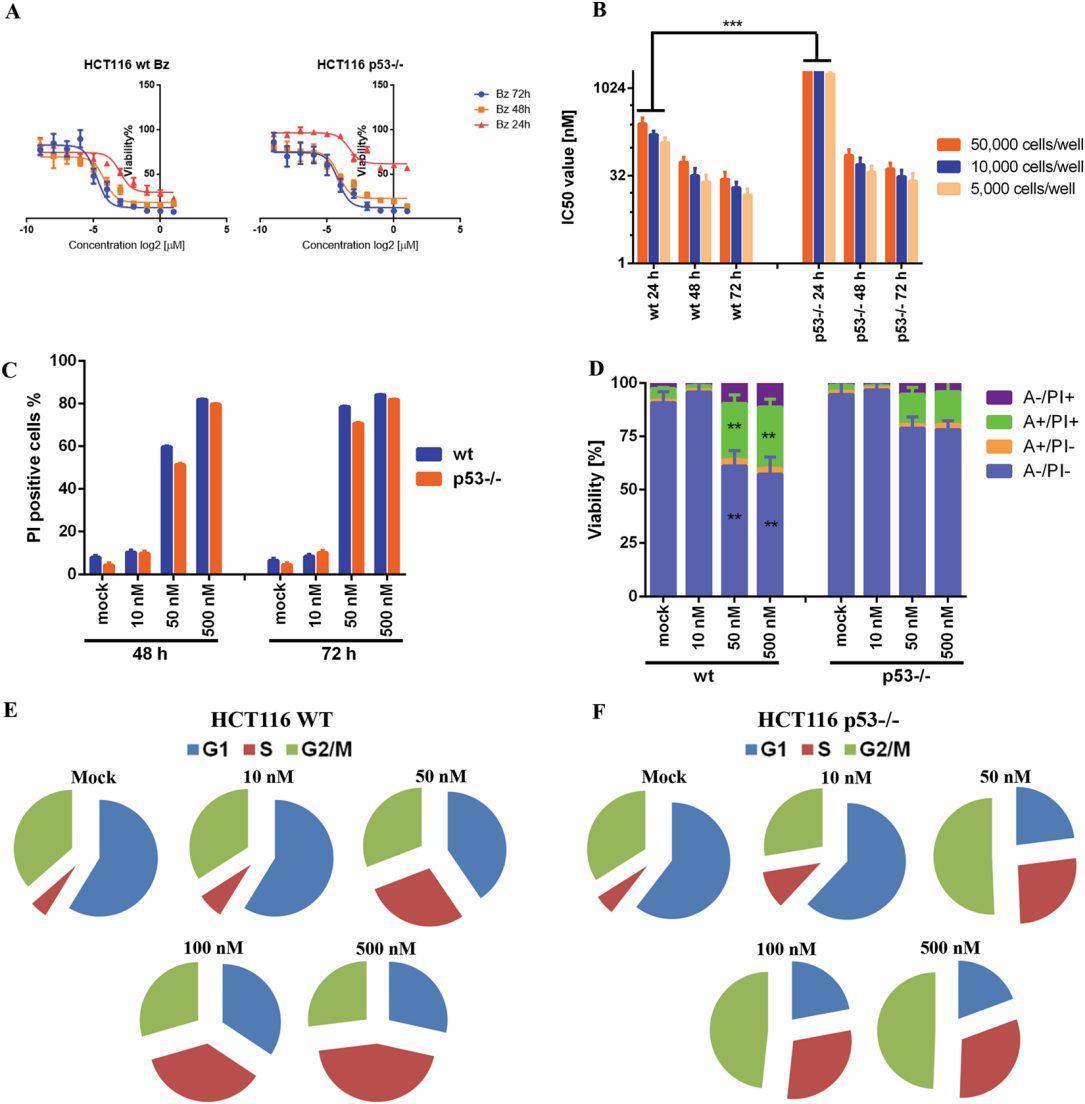
Transient resistance to bortezomib in HCT116 p53−/− cells. (**A**) Anti-proliferative effect of bortezomib on HCT116 wt and p53−/− cells in MTT assay. The cells were treated with increasing concentrations of bortezomib for 24 hrs, 48 hrs and 72 hrs at a seeding density of 10,000 cells/well. Cell viability was determined by the number of treated over control cells (0.5% DMSO). The software ‘Prism’ was used to obtain dose-response curves. (**B**) The comparison of IC50-values [nM] in HCT116 wt and p53−/− cells treated with bortezomib for 24 hrs, 48 hrs and 72 hrs at three initial densities of 5,000, 10,000 and 50,000 cells/well. IC50-value [nM] was calculated from dose-response curves. (**C**) the number of dead cells was comparable in both cell lines in long-term treatment. HCT116 wt and p53−/− cells were treated with bortezomib (10 nM, 50 nM and 500 nM) for 48 hrs and 72 hrs. The percentage of dead cells labeled with propidium iodide (PI) was analyzed by FACS. DMSO (final concentration: 0.5%) was used as mock. (**D**) Bortezomib induced apoptosis in HCT116 wt and p53−/− cells after 24 hrs treatment. Cells were treated with increasing concentrations of bortezomib as indicated for 24 hrs and labeled with annexin v to visualize apoptotic cells, PI for necrotic cells and double staining for apoptotic cells at late stage. (**E** and **F**) Bortezomib induced cell cycle arrest in HCT116 wt and p53−/− cells. HCT116 wt and p53−/− cells were treated with increasing concentrations of bortezomib (0.01 µM, 0.05 µM, 0.1 µM and 0.5 µM) for 24 h. 0.5% DMSO was used as mock treatment. DNA content was studied by staining with PI after ethanol fixation. Comparable results were obtained from at least three independent experiments. One was depicted for (**C**,**D**,**E** and **F**). *P < 0.05; **p < 0.01 and ***p < 0.001, Bz: bortezomib.

Previous reports have shown contradicting results regarding the resistance of HCT116 p53−/− cells to bortezomib^24–28^. To address this controversy, we re-evaluated the anti-proliferative effect of bortezomib in HCT116 wt and p53−/− cells at three seeding densities, 5,000, 10,000 and 50,000 cells/well, and three incubation time points, 24, 48 and 72 hrs, using MTT viability assay. As expected, IC50-values, calculated from dose-response curves, were inversely related to incubation times (Fig. 1A), while positively to initial cell numbers (Fig. 1B). In comparison to those in wt cells nearly 10-fold higher IC50-values were found in p53−/− cells within 24 hrs treatment regardless of the seeding density (Fig. 1A and B), while became comparable (less than 2-fold) after 48 hrs or 72 hrs treatments (Fig. 1A and B). Moreover, quantitative FACS (fluorescence activated cell sorting) analysis clearly showed similar amounts of dead cells at all tested concentrations labeled with PI (propidium idodide) after 48 hrs or 72 hrs incubation (Fig. 1C), confirming that the resistance to bortezomib in p53−/− cells was transient and occurred within 24 hrs.

### Comparable cellular responses in HCT116 wt and p53−/− cells to bortezomib

The observation of transient p53-dependent resistance in HCT116 cells implicated the existence of a substitute to trigger apoptosis in the absence of p53. We first studied bortezomib-induced cell death in an apoptotic assay using annexin v/PI staining^29, 30^ and confirmed that apoptosis occurred in both cell lines within 24 hrs of treatment (Fig. 1D). However, number of dead cells in HCT116 wt was clearly higher than that in p53-deficeient cells (Fig. 1D). Analysis of the DNA content upon treatment showed that S-phase arrest played a dominant role in wt cells (Fig. 1E), while S- and G2/M-phase arrests were found in p53−/− cells (Fig. 1F).

**Figure 2:**
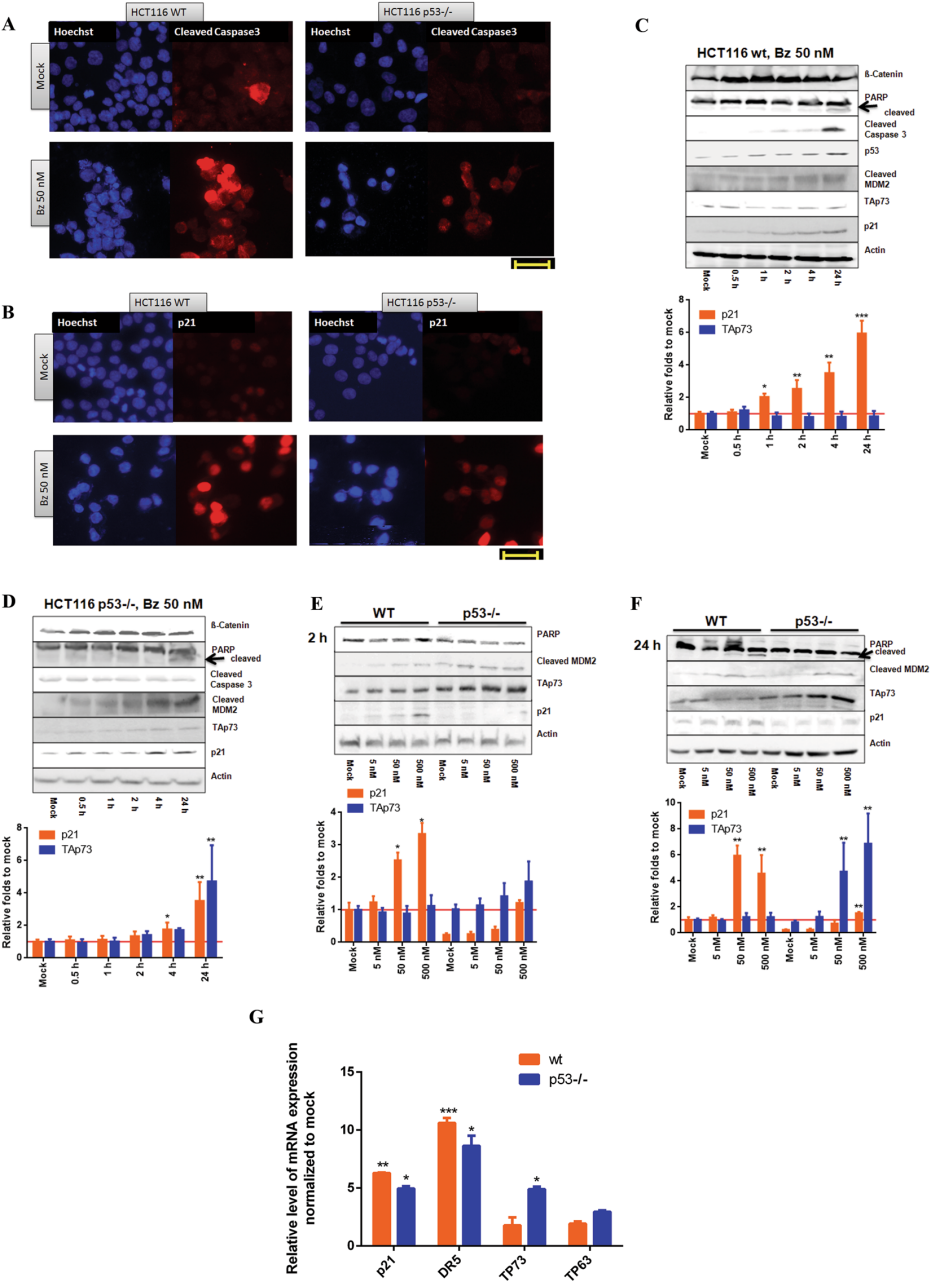
Similar cellular responses to bortezomib in HCT116 wt and p53−/− cells within 24 hrs. (**A**) Bortezomib induced cleavage of Caspase 3 in HCT116 wt and p53−/− cells. After treatment with bortezomib (50 nM) for 24 hrs, immunocytochemistry was employed to compare the presence of cleaved Caspase3 by using specific antibody. Hoechst dye was used to indicate nuclei. Scale bar: 40 µm. DMSO (final concentration: 0.5%) was used as mock. (**B**) Bortezomib activated p21 in HCT116 wt and p53−/− cells. Scale bar: 40 µm. DMSO 0.5% was used as mock. (**C**) Study of time-dependent cellular responses analyzed by immunoblotting in HCT116 wt cells and (**D**) in HCT116 p53−/− cells. The whole cell lysate was subjected to immunoblotting. Specific antibodies as indicated were used. ß-Actin served as loading control. (**E**) Concentration-dependent analysis upon treatment in HCT116 wt and p53−/− cells after 2 hrs and (**F**) 24 hrs treatment with bortezomib. (**G**) qRT-PCR analysis on the expression of members of the p53 family and their downstream genes. Cells were treated with a concentration of 50 nM of bortezomib for 24 hrs. 0.5% DMSO was used as mock. Densitometric analyses were conducted by using Aida software (Raytest). *P < 0.05; **p < 0.01 and ***p < 0.001.

Compelling evidence provided the important roles of Caspase 3 and p21 for inducing apoptosis in HCT116 cell lines^31–35^. We compared the levels of cleaved Caspase 3 and p21 in treated wt and p53−/− cells using fluorescence microscopy. As showed in Fig. 2A and B, cleaved Caspase 3 and p21 could be detected in both cells upon 24 hrs of bortezomib treatment. However, the intensities in wt cells were much higher than those in p53−/− HCT116 cells.

In a time-dependent study, accumulation of ß-Catenin occurred as early as 30 min in both cells, implicating an immediate effect of bortezomib on the ubiquitin proteasome pathway. Since apoptosis is a time-consuming process of programmed cell death^30^, the clear cleavage of PARP, a hallmark of apoptosis^36^, appeared after 24 hrs (Fig. 2C and D). As expected, p53 was expressed only in wt cells and accumulated over the test period (Fig. 2C). Moreover, cleaved Caspase 3 was observed in HCT116 wt as early as 2 hrs treatment (Fig. 2C), while undetectable in p53−/− cells after 24 hrs treatment (Fig. 2D), reflecting a much weaker induction of Caspase 3 cleavage in HCT116 p53−/−, that was not sufficient to be detected in immunoblotting using the whole cell lysate. Interestingly, the constant enrichment of cleaved MDM2, a positive indicator of p53 stability^37^, was found in both cell lines (Fig. 2C–F). In good agreement to the previous report^37^, accumulation of p21 was observed in wt cells as early as 1 hrs upon treatment (Fig. 2C). Interestingly, activation of p21 was also detected in cells lacking p53 upon 4 hrs treatment (Fig. 2D).

To investigate if p53-associated signaling pathways were activated upon treatment, we analyzed the expression of p21 and DR5 by qRT-PCR and found that these two well-known p53 target molecules^32, 34^ were induced up to 6 and 10 folds in HCT116 wt cells respectively (Fig. 2G), suggesting that transcriptional induction of p21 at least partially contributed to the observed p21 accumulation mediated by bortezomib except for its direct inhibition of p21 protein degradation. Again, the activation of p21 and DR5 could be detected in p53−/− cells, showing 5- and 8-fold higher induction compared to DMSO treatment as mock (Fig. 2G).

### TAp73 as a substitute for p53 to induce apoptosis in HCT116 p53−/− cells

**Figure 3:**
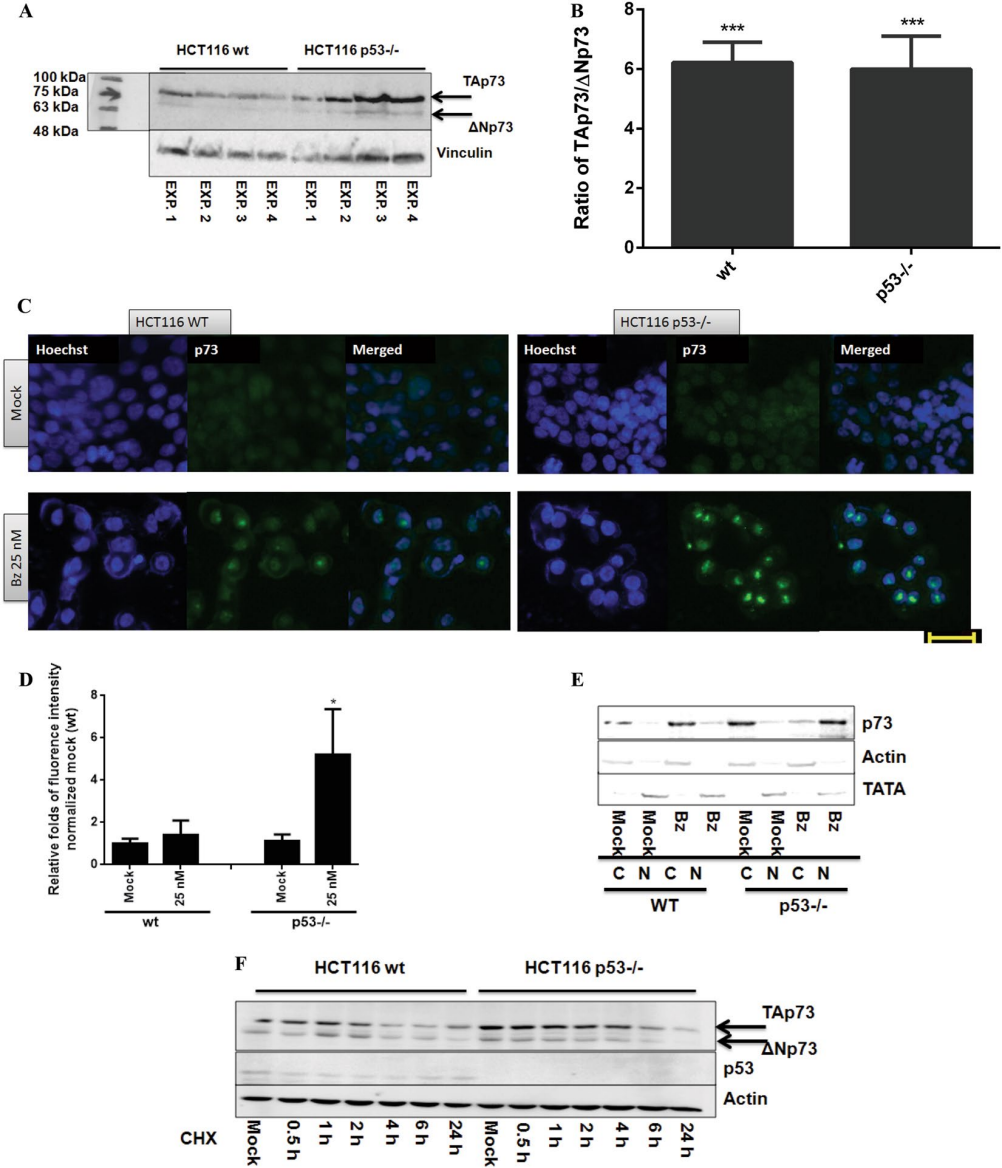
TAp73 represents as the majority of p73 protein expression in HCT116 wt and p53−/− cells. (**A**) Two isoforms, TAp73 (85 kDa) and ΔNp73 (65 kDa), were reproducibly detected in HCT116 and p53−/− cells. Vinvulin was used as loading control. (**B**) Densitometric analysis on the ratio of TAp73 and ΔNp73 expression. (**C**) Translocation of p73 to the nucleus upon treatment analyzed by immunocytochemistry. Scale bar: 40 µm. DMSO (final concentration: 0.5%) was used as mock. (**D**) The quantification of fluorescence intensity, which is described in detail in the method section. (**E**) Nuclear accumulation of TAp73 occurred in the presence of bortezomib (50 nM) for 24 hrs detected by using separated nuclear and cytosolic fractions in wt and p53−/− cells (C: cytosolic fraction, N: nuclear fraction). (**F**) p73 expression in the presence of cyclohexamide (CHX). HCT116 wt and p53−/− cells were treated with 10 µM CHX, collected at 0.5 h, 1 h, 2 h, 4 h, 6 h and 24 h and subjected to immunoblotting. ß-Actin was used as loading control. *P < 0.05 and ***p < 0.001.

High similarities in wt and p53−/− cells in response to bortezomib, including MDM2 cleavage, p21 stabilization and DR5 induction let us to assume that other member(s) of the p53 family might be activated and took over the function of p53 to induce apoptosis in p53-deficient cells^10^. We analyzed the expression of two structurally and functionally related p53 homologues, p63 and p73. In good agreement with the previous study^34^, we observed the expression of p73 in HCT116 cells (Fig. 3A), but p63 was undetectable (data is not shown). Previous studies reported a molecular weight of 85 kDa for TAp73 and 65 kDa for the amino-terminal lacking counterpart, ΔNp73^38^. We densitometrically analyzed the expression of TAp73 and ΔNp73 in HCT116 wt and p53−/− by immunoblotting on the basis of their clearly distinguishable molecular weights. As shown in Fig. 3B, TAp73 was the major isoform of p73 in both cell lines, exhibiting nearly 6-fold higher expression than ΔNp73.

Focusing on the function of TAp73, its cellular level in HCT116 p53−/− after 24 hrs treatment with bortezomib was remarkably elevated at 50 nM and 500 nM concentrations to induce apoptosis (Fig. 2D and F), while kept almost unaffected in the presence of p53 (Fig. 2C and F). Analysis on the transcriptional level, confirmed the induction of TP73 in response to bortezomib, showing approximately 5-fold higher expression in comparison to DMSO treatment in cells lacking p53, while remaining unchanged in HCT116 wt (Fig. 2G). Since the transcriptional activity of p73 is associated with its translocation from cytoplasm to the nucleus, we carried out the immunocytochemistry method and detected nuclear accumulation of p73 in p53−/− cells treated with bortezomib. The intensity was remarkably higher than that in wt cells (Fig. 3C and D). To further confirm the nuclear retention of p73 in p53−/− HCT116 cells, we separated nuclear and cytosolic fractions and performed immunoblotting, showing that p73 nuclear accumulation upon bortezomib treatment occurred only in the absence of p53 (Fig. 3E). Moreover, we compared the expression levels of p73 in wt and p53−/− cells using the translation inhibitor, cycloheximide (CHX)^21^. We observed a higher stability of p73 in p53-deficient cells compared to wt cells regardless of the bortezomib treatment (Fig. 3F).

### Enhanced resistance in HCT116 p53−/− p73KD and HT-29 p73KD cells

**Figure 4:**
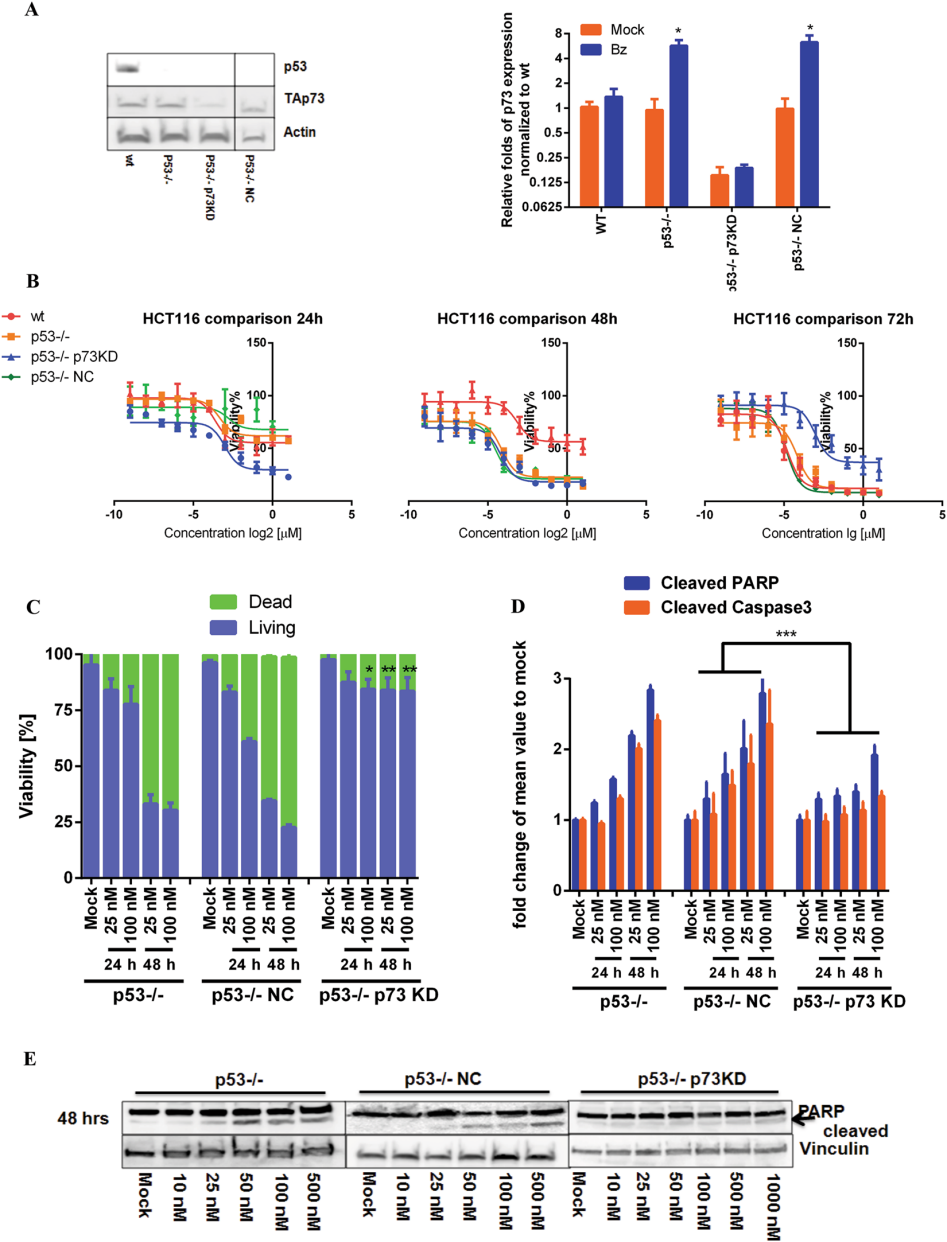
An essential role of p73 for replacing p53 in bortezomib-induced apoptosis in HCT116 cells. (**A**) Knockdown efficiency of TAp73 by CRISPR/Cas9. The whole cell lysates of the stabilized knockdown cell line, named HCT116 p53−/− p73KD, were subjected to immunoblotting. The efficiency was evaluated by densitometric quantification of immunoblotting results. qRT-PCR analysis on the expression of p73 in all the four cell lines in the absence/presence of bortezomib (50 nM) for 24 h. The expression of p73 was normalized to mock treatment (DMSO 0.5%) in HCT116 wt cells. HCT116 p53−/− cells were transfected with non-targeting gRNA (HCT116 p53−/− NC), thereafter used as negative control. (**B**) Anti-proliferative effect of bortezomib in HCT116 p53−/− p73KD in comparison to that in wt, p53−/− and p53−/− NC cells after 24 hrs, 48 hrs and 72 hrs treatments. (**C**) Viability of p73KD cells was compared to that of p53−/− and p53−/− NC cells labeled with PI after 24 hrs and 48 hrs treatments. DMSO (final concentration: 0.5%) was used as mock. (**D**) Cleavage of PARP and Caspase 3 was compared in HCT116 p53−/−, p53−/− NC and p73KD cells stained with specific antibodies and analyzed by FACS. 0.5% DMSO was used as mock. (**E**) The cleavage of PARP was studied in HCT116 p53−/−, p53−/− NC and p73KD cells treated with increasing concentrations of bortezomib analyzed by immunoblotting. DMSO 0.5% was used as mock. *P < 0.05; **p < 0.01 and ***p < 0.001.

The above results apparently suggested that TAp73 served as a substitute for p53 in response to bortezomib in p53-deficient HCT116 cells. To further validate p73 as the primary modulator in bortezomib-induced apoptosis, we knocked down the expression of TP73, using a commercially available CRISPR/Cas9 plasmid against TP73 and achieved the repression down to 80% detected by immunoblotting (Fig. 4A), thereafter referred to as HCT116 p53−/− p73KD cells. Additionally, we included p53−/− cells transfected with a non-targeting CRISPR/Cas9 vector named as HCT116 p53−/− NC (negative control). We evaluated the anti-proliferative effects of bortezomib in this newly generated, stable cell line. The results interestingly showed comparable IC50-value to that in p53−/− cells in 24 hrs treatment (Fig. 4B, left), whereas the resistance was notably increased in p73KD cells in 48 hrs (Fig. 4B, mid) and 72 hrs treatments (Fig. 4B, right). Of note, similar levels of p73 expression were observed in non-treated wt and p53−/− HCT116 as well as p53−/− NC cells, while bortezomib-induced activation of p73 appeared only in p53−/− and p53−/− NC cells (Fig. 4A). FACS analysis confirmed that populations of dead cells treated with bortezomib for 24 hrs were similar in p53−/−, p53−/− NC and p73KD cell lines (Fig. 4C), whereas almost 3-fold higher in p53−/− and p53−/− NC cells than in p73KD cells upon 48 hrs treatment (Fig. 4C). Moreover, bortezomib time- and concentration-dependently induced cleavage of PARP and Caspase 3 in p73KD cells (Fig. 4D), although the intensity was much less in comparison to those in p53−/− and p53−/− NC cells (Fig. 4D). Consistently, PARP cleavage was clearly detectable in p53−/− and p53−/− NC cells by immunoblotting at concentrations above 25 nM after 48 hrs treatment, but not in HCT116 p73KD cells (Fig. 4E).

**Figure 5:**
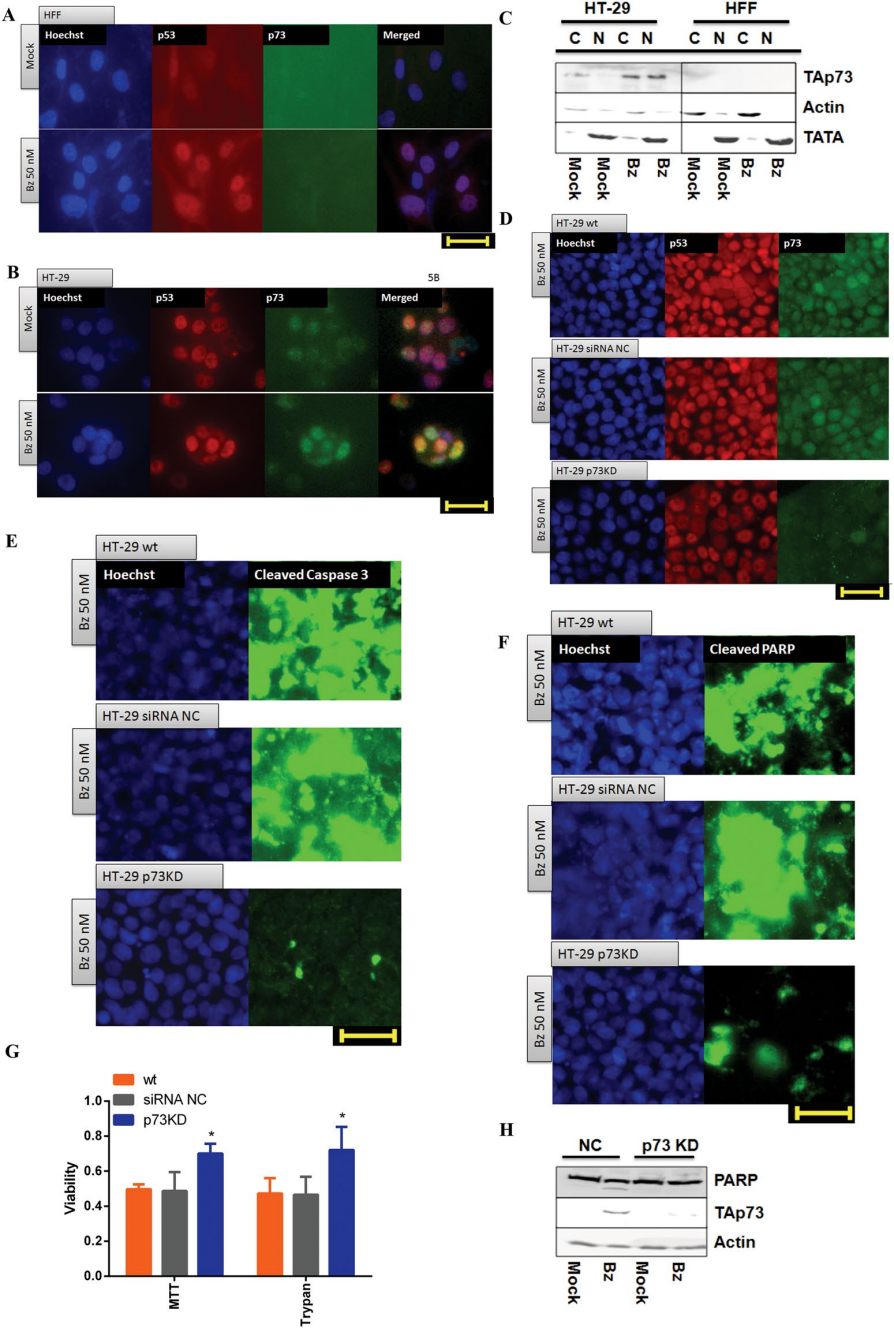
p73-deficient HT-29 cells are refractory to bortezomib. (**A**) 24 hrs treatment of bortezomib (50 nM) did not cause p73 nuclear accumulation in human fibroblast (HFF) detected by p53 (red) and p73 (green) antibodies in immunoncytochemistry. Scale bar: 40 µm. DMSO 0.5% was used as mock. (**B**) Nuclear accumulation of p73 appeared upon bortezomib treatment (50 nM for 24 hrs) in HT-29 cells. Scale bar: 40 µm. DMSO 0.5% was used as mock. (**C**) Bortezomib (50 nM) induced p73 nuclear accumulation in HT-29 cells after 24 hrs but not in HFF, using separated subcellular extracts. Final concentration of 0.5% DMSO was used as mock. (**D**) Comparison of p73 translocation to the nucleus upon bortezomib treatment (50 nM for 24 hrs) between HT-29, HT-29 transfected cells with non-targeting siRNA (HT-29 siRNA NC) as negative control, and HT-29 p73 knockdown cells (HT-29 p73KD). Scale bar: 40 µm. (**E**) Reduced cleaved Caspase 3 in HT-29 p73KD in contrast to its parental cell line and HT-29 siRNA NC after 24 hrs treatment of bortezomib (50 nM) Scale bar: 40 µm. (**F**) Reduction of cleaved PARP in HT-29 p73KD, compared to HT-29 and HT-29 siRNA NC cells upon 24 hrs of bortezomib treatment (50 nM). Scale bar: 40 µm. (**G**) Evaluation of cell viability in HT-29 p73KD cells and its wild-type counterpart as well as HT-29 siRNA NC. Cells were treated with bortezomib (50 nM) for 48 hrs. Cell viability was measured by MTT and trypan blue assays. The data were normalized to the respective mock treatment (DMSO 0.5%). (**H**) Analysis of p73 and cleaved PARP expression by immunoblotting upon 48 hrs treatment of bortezomib (50 nM). ß-Actin was used as loading control. HT-29 siRNA NC and p73KD cells were treated with 50 nM of bortezomib for 48 hrs. 0.5% DMSO was used as mock. *P < 0.05.

Finally, we investigated the role of p73 in bortezomib-induced apoptosis in human foreskin fibroblast (HFF) cell line, which expresses functional p53, as well as HT-29, carrying mutated p53. In good agreement with the observation in HCT116 wt, the nuclear accumulation of p53 was found in HFF upon bortezomib treatment, but not p73 (Fig. 5A), whereas nuclear localization of p73 was evident in HT-29 cells by analyzing nuclear and cytosolic fraction (Fig. 5C), as well as by immunocytochemistry (Fig. 5B and D). Correspondingly, we detected remarkable cleavage of Caspase 3 (Fig. 5E) and PARP (Fig. 5F), both of which nearly disappeared in HT-29 p73KD achieved by using a p73 siRNA (Figs. 5E and F), in which non-targeting siRNA was used as a negative control (HT-29 siRNA NC). The results from MTT and trypan blue assays confirmed higher number of living cells after 48 hrs treatment of bortezomib in HT-29 p73KD cells compared to its parental cell line (Fig. 5G). In line with the viability tests, PARP cleavage was barely detectable in p73-deficient HT-29 cells after 48 hrs treatment of bortezmib (Fig. 5H).

## Discussion

The proteasome inhibitor bortezomib is currently used in the treatment of multiple myeloma. Besides, several clinical and preclinical studies have shown the drug to be a promising candidate against other malignancies including colorectal cancer^13–16, 39^. Despite the initial remission, bortezomib-therapy seems to fail eventually due to the inevitable drug-resistance. Cellular mechanisms that confer the resistance to bortezomib, mainly target its mode of action, including inherent mutations in PSMB5 and up regulation of proteasome subunits^23^. Furthermore, down-regulation of the key apoptotic molecule p53 and its target genes, including p21, has been shown to attenuate bortezomib efficacy, confirming the p53-dependent mechanism of the drug^40^. Here we show for the first time a crucial role of p73, a p53-related protein, in either p53-deficient or mutant p53 harbouring colon cancer cell lines in response to proteasomal inhibition by bortezomib.

p73 unlike its close homologue, p53, is rarely mutated in cancer^41^. However, the transcriptionally active isoform of the protein, TAp73 is frequently inhibited by the dominant-negative effect of the N-terminal domain lacking variant, ΔNp73. Thus, the relative ratio of TA/ΔN appears to be a determinant of p73-mediated apoptosis, cell cycle arrest and chemotherapeutic sensitivity. In this regard, several reports have shown the importance of p73 status in anti-cancer drug efficacy and clinical outcome of patients. p73 has been shown to be involved in the apoptotic response of the DNA-damaging agents, such as cisplatin and adriamycin in different cancerous cell lines^42, 43^. Over-expression of TAp73 enhances the pro-apoptotic effects of bleomycin, mitoxantrone and doxorubicin while ΔNp73 up-regulation has been associated with drug-resistance and poor prognosis in hepatocellular carcinoma^12^. Besides, it has been reported that down regulation of mutant p53 reduces chemo-resistance partly through restoring the tumor suppressor activity of TAp73, affirming that some p53 mutations exert their effects by blocking the pro-apoptotic function of p73^44^.

In this study, we report transient resistance to bortezomib cytotoxicity in HCT116 p53−/− cells in comparison to its parental cell line HCT116 wt. However in long term treatment, the IC50 values and the number of dead cells became comparable between two cell lines. Further analysis revealed similar regulation of apoptosis-associated signalling molecules by bortezomib in both cell lines, including activation of p53 responsive genes and the cleavage of caspase 3 and PARP, however to a lesser degree in p53-deficient cells. Given the similarity in responses to bortezomib in the different cell lines, we hypothesized that other members of the p53 family could be activated in response to treatment in order to compensate for the lost p53 function. p63 and p73 are members of the p53 family^10, 45^, whose transcriptionally active isoforms have been identified as tumor suppressors that induce apoptosis and cell cycle arrest under various conditions^46^. TAp73 represented the major isoform in HCT116 cells, showing nearly 6 fold higher expression than ΔNp73. This is consistent with the previous finding that aberrant expression of p63 was found only in germ cells of the ovary and testis^47^. Upon treatment, we detected a clear induction, stabilization and nuclear translocation of TAp73 solely in cells with absent or mutated p53, HCT116 p53−/− and HT29 cells, respectively, but not in cells harbouring wild type p53, which is in line with previous reports demonstrating a reduced competitive capacity of TAp73 to functional p53^10^.

Despite the known anti-tumorigenic effects of TAp73 *in vitro* and *in vivo* ^10, 46^, it has been suggested in individual reports that TAp73 could facilitate malignant transformation in MEF cells by either promoting NADPH synthesis^12^ or by activating angiogenic signaling pathways under hypoxia^48^. Thus, In an effort to further confirm the pro-apoptotic role of TAp73 in different experimental settings, we used a CRISPR/Cas9 plasmid against TP73 or siRNA-mediated down regulation of the p73 mRNA, both of which achieved sufficient repression in HCT116 and HT-29 cells, respectively. As expected, we could retain the resistance to bortezomib upon disrupting the transcriptional activity of TAp73 over the test period.

We therefore, conclude that TAp73 substitutes p53 in cells lacking functional p53, mediating the delayed pro-apoptotic effects of bortezomib. The efficacy however was lower in comparison to that mediated by p53 in the first 24 hrs. TAp73 deletion indeed led to a sustained resistance, highlighting the important role of the molecule in bortezomib chemo-sensitivity. Since bortezomib is widely used in clinical settings, it is very important to translate the present work to primary tissues by including patient samples. Up-regulation of the amino-terminal deleted isoform ΔNp73 has been shown to predispose patients to cancer^12^. Therefore, the ability of TAp73 to take over the tumor-suppressive effects of p53 in response to bortezomib, in colorectal cancer patients with mutated p53 could be an interesting and clinically relevant future step to the current project.

## Methods

### Chemicals and Antibodies

Bortzemib was purchased by Selleck (Germany). Primary antibodies for western blot; ß-Catenin (9582), p53 (9282 and 2527), Caspase 3 (9662), PARP (9542), p21 (2947), cleaved PARP (5625) and cleaved Caspase 3 (9664) were obtained from cell signaling (NEB, Germany). p73 antibody (14620), capable of recognizing both isoforms of p73, was also purchased form cell signalling (NEB, Germany). MDM2 (SC-812), actin (SC-47778), p53 (SC-126) and Vinculin (SC-73214) were from Santa Cruz (Germany).

### Cell culture

HT-29, HFF and HCT116 were cultured in DMEM + GlutaMAX-1 (Ref# 61965-026, Gibco, Germany) containing 10% FBS (Ref# 15140-12, Gibco) and 1% penicillin/streptomycin (Ref# 15140-12, Gibco, Life technologies, Germany) under 5% CO_2_ at 37 °C in a humidified atmosphere.

### Generation of HCT116 p53−/− p73 knockdown cells (HCT116 p53−/− p73KD)

CRISPR/Cas9 p73 plasmid (SC-416822-NIC) was purchased from Santa Cruz (Germany). The sequences of gRNA are tggatgaccctgtcaccggc for strand A and tattgccttccacgcggatg for strand B. Non-targeting gRNA was used as negative control (SC-437281). Lipofectamine 3000 (Life Technologies, Germany) was used for transfection. According to the manufacturer’s instruction, transfection was carried out in a 24-well plate with seeding density of 200,000 cells/well. A mixture of 0.5 µg/well DNA and Lipofectamine 3000 was added and incubated for 24 hrs. For the selection 0.5 µg/mL puromycin was used. The established HCT116 p53−/− p73KD cell line was cultivated in DMEM with 10% FCS, 1% PS and 0.5 µg/mL puromycin. The medium was changed two times per week.

### p73 siRNA transfectin

p73 siRNA was purchased from Thermofisher Scientific (115666) and transfection was performed according to the manufacturerr’s instruction at a final concentration of 20 nM for the p73 siRNA. Non-targeting siRNA was used as negative control^21^. Lipofectamine 3000 was used as transfection reagent. The treatment was carried out 24 hrs after transfection as indicated in the text.

### MTT assay

3-(4,5-Dimethylthiazol-2-yl)-2,5-diphenyl-2H-tetrazolium bromide (MTT) assay was performed to determine the anti-proliferative effects of compounds as previously reported^30^. Briefly, cells were plated into 96-well plates at a density of 50,000, 10,000 and 5,000 cells/cm^2^ and incubated for 24 hrs. The cells were treated with increasing concentrations of bortezomib in complete medium in quadruplicate. After 24 hrs, 48 hrs and 72 hrs, the culture medium was replaced with a solution of MTT (25 mg/50 mL) in medium containing 1% FBS, incubated for 2 hrs and quantified photometrically at 595 nm. Cytotoxicity was calculated as percent survival, determined by the number of treated cells over mock. The comparable results were obtained from three independent experiments.

### Trypan blue assay

The cells were cultivated in DMEM (10% FBS), transfected with siRNAs and treated with bortezomib. The cells were trypsinized and re-suspended in medium. A mixture of 20 µL cell suspension and 20 µL trypan blue was added into a hemocytometer chamber. The number of cells was scored under the microscope and was normalized to the corresponding mock treatment.

### Apoptosis assay

HCT116 cells were incubated with compounds as indicated. The cells were trypsinized, resuspended in 50 µL annexin V binding buffer and incubated with 5 µL FITC-conjugated annexin V (BD Bioscience, Germany) for 15 min in the dark at room temperature. Afterwards, the suspension was diluted in 450 µL annexin V binding buffer containing 1.25 µL propidium iodide (PI, 1 mg/mL), incubated for 10 min in the dark at room temperature and analyzed with FACS as described^12, 29^. DMSO (final concentration: 0.5%) was used as mock. The comparable results were obtained from at least three independent experiments.

### Immunoblotting

Cell extracts were homogenized in urea-lysis buffer (1 mM EDTA, 0.5% Triton X-100, 5 mM NaF, 6 M Urea, 1 mM Na_3_VO_4_, 10 µg/mL Pepstatin, 100 µM PMSF and 3 µg/mL Aprotinin in PBS). The blots were detected with ECL solution as previously reported^29, 30^. 40 µg of total protein was resolved on 10% SDS-PAGE gels and immunoblotted with specific antibodies. Primary antibodies were incubated at a 1:1,000 dilution in blocking buffer with gentle agitation overnight at 4 °C. The comparable results were obtained from at least three independent experiments. Densitometric analyses of corresponding immunoblots were conducted using Aida software (Raytest) according to the manufacturer’s instruction

### Immunofluorescence

As we described before^49, 50^, HCT116 cells were seeded in a Ø-12mm cover slip coated with Geltrex (Life Technologies, Germany) at a density of 200,000 cells/well. After 24 hrs the cells were incubated with bortezomib at designed concentrations for 24 hrs, fixed with 4% PFA at RT (room temperature) for 15 min, and blocked with blocking buffer (5% goat serum, 1% BSA and 0.3% Triton X-100 in PBS) for 30 min. The blocking solution was aspirated and incubated with p53 (1:200), cleaved Caspase3 (1:200), p21 (1:200) or p73 (1:200) in blocking buffer at RT for 1 hrs. The secondary antibody (Goat anti-rabbit Alexa Flor 488 and Goat anti-mouse Alexa Flor 594; Dianova) was added and incubated for 30 min. Hoechst 33342 (1 µg/mL in PBS) was used to visualize nuclei. Images were taken on BIOREVO fluorescence microscope (BZ9000, KEYENCE). DMSO (final concentration: 0.5%) was used as mock. The quantification of fluorescence intensity was performed by the software Image J according to the manufacturer’s instructions. The analyzed images were randomly selected and counted from 10 fields.

### Cell cycle analysis

HCT116 cells were treated with bortezomib. After trypsinization, the pellets were fixed in 70% Ethanol and stored for at least 24 hrs at −20 °C, were washed two times with ice-cold PBS, were incubated with RNAase (200 µg/mL) for 30 min at 37 °C and with PI (50 µg/mL) for 30 min at room temprature. The mixture was washed with PBS and analyzed with FACS as described^29, 30^. DMSO (final concentration: 0.5%) was used as mock. The comparable results were obtained from three independent experiments. One experiment is depicted.

### Primers for qRT-PCR

Quantitative real-time reverse transcription-PCR was performed according to manufacturing instruction (qTower, Analytik Jena, Germany). Briefly, total RNA was isolated from cells using RNeasy kit from Qiagen. cDNA was generated by reverse-transcription of equivalent quantities of RNA using ProtoScript® II first strand cDNA synthesis kit (New England Biolabs, Germany) and qRT-PCR was performed using SYBR Green PCR master mix on qTower (Analytik Jena)^29^. The primers were purchased from Eurofins (Germany) and the sequences were listed below. Actin was used as an endogenous control. p21 (5s: gacaccactggagggtgact; 3as: caggtccacatggtcttcct), DR5 (5s: gagctaagtccctgcaccac; 3as: ccccactgtgctttgtacct), p73 (5s: catggagacgaggacacgta; 3as: gtgactcggcctctgtagga); p63 (5s: gaaacgtacaggcaacagca; 3as: gctgctgagggttgataagc). Actin (5s: ctgactacctcatgaagatcctc; 3as: cattgccaatggtgatgacctg)

### Flow cytometry analysis of immunostaining

HCT116 cells were incubated with bortezomib as designed. The cells were trypsinized, resuspended in PBS, fixed with 4% PFA and blocked in blocking buffer for 1 hrs^49^. The suspension was incubated with either cleaved PARP or caspase 3 over night. Afterwards, the suspension was washed, incubated with secondary antibody (Goat anti-rabbit Alexafluor 488-conjugated) for 1 hrs and analyzed with FACS as described above. DMSO (final concentration: 0.5%) was used as mock.

### Subcellular fractionation

Trypsinized cells were washed with PBS, lysed in a lysis buffer containing 250 mM sucrose, 20 mM HEPES (pH 7.4), 10 mM KCl, 1.5 mM MgCl_2_, 1 mM EDTA and 1 mM EGTA in PBS. 1 mM DTT, 5 mM NaF, 1 mM Na_3_VO_4_, 10 µg/mL pepstatin, 100 µM PMSF and 3 µg/mL aprotinin were freshly added and were passed through a 25 G needle 20 times. Nuclear pellet was obtained by centrifuging at 720 g for 5 min and was washed once with the lysis buffer. Supernatant was centrifuged at 10,000 g for 20 min and was washed once with the lysis buffer to yield the cytosolic fraction.

### Statistical analysis

The statistical significance of compared measurements was performed using the Student’s one-tailed or two-tailed t-test (Microsoft Excel).

### Data Availability

Authors declare that all the data supporting the findings of this study are included within the article.

## References

[1] Brenner H, Kloor M, Pox CP (2014). Colorectal cancer. Lancet.

[2] Markowitz SD, Bertagnolli MM (2009). Molecular origins of cancer: Molecular basis of colorectal cancer. N Engl J Med.

[3] Fearon ER (2011). Molecular genetics of colorectal cancer. Annu Rev Pathol.

[4] Aklilu M, Eng C (2011). The current landscape of locally advanced rectal cancer. Nat Rev Clin Oncol.

[5] Vogelstein B, Lane D, Levine AJ (2000). Surfing the p53 network. Nature.

[6] Khoo KH, Verma CS, Lane DP (2014). Drugging the p53 pathway: understanding the route to clinical efficacy. Nat Rev Drug Discov.

[7] Liu Y (2015). TP53 loss creates therapeutic vulnerability in colorectal cancer. Nature.

[8] Muller PA, Vousden KH (2014). Mutant p53 in cancer: new functions and therapeutic opportunities. Cancer Cell.

[9] Iacopetta B (2003). TP53 mutation in colorectal cancer. Hum Mutat.

[10] Melino G, De Laurenzi V, Vousden KH (2002). p73: Friend or foe in tumorigenesis. Nat Rev Cancer.

[11] Kartasheva NN, Contente A, Lenz-Stoppler C, Roth J, Dobbelstein M (2002). p53 induces the expression of its antagonist p73 Delta N, establishing an autoregulatory feedback loop. Oncogene.

[12] Tannapfel A (2008). Autonomous growth and hepatocarcinogenesis in transgenic mice expressing the p53 family inhibitor DNp73. Carcinogenesis.

[13] Adams J (2004). The development of proteasome inhibitors as anticancer drugs. Cancer Cell.

[14] Adams J (2002). Development of the proteasome inhibitor PS-341. Oncologist.

[15] Rajkumar SV, Richardson PG, Hideshima T, Anderson KC (2005). Proteasome inhibition as a novel therapeutic target in human cancer. J Clin Oncol.

[16] Lenz HJ (2003). Clinical update: proteasome inhibitors in solid tumors. Cancer Treat Rev.

[17] Niewerth D (2015). Molecular basis of resistance to proteasome inhibitors in hematological malignancies. Drug resistance updates: reviews and commentaries in antimicrobial and anticancer chemotherapy.

[18] Williams SA, McConkey DJ (2003). The proteasome inhibitor bortezomib stabilizes a novel active form of p53 in human LNCaP-Pro5 prostate cancer cells. Cancer research.

[19] MacLaren AP, Chapman RS, Wyllie AH, Watson CJ (2001). p53-dependent apoptosis induced by proteasome inhibition in mammary epithelial cells. Cell death and differentiation.

[20] Mitsiades CS (2006). Antitumor effects of the proteasome inhibitor bortezomib in medullary and anaplastic thyroid carcinoma cells *in vitro*. J Clin Endocrinol Metab.

[21] Cheng X (2012). Indirubin derivatives modulate TGFbeta/BMP signaling at different levels and trigger ubiquitin-mediated depletion of nonactivated R-Smads. Chem Biol.

[22] Strauss SJ (2007). The proteasome inhibitor bortezomib acts independently of p53 and induces cell death via apoptosis and mitotic catastrophe in B-cell lymphoma cell lines. Cancer research.

[23] Adams J (1999). Proteasome inhibitors: a novel class of potent and effective antitumor agents. Cancer Res.

[24] Chen S (2010). Genome-wide siRNA screen for modulators of cell death induced by proteasome inhibitor bortezomib. Cancer research.

[25] Roger L, Jullien L, Gire V, Roux P (2010). Gain of oncogenic function of p53 mutants regulates E-cadherin expression uncoupled from cell invasion in colon cancer cells. J Cell Sci.

[26] Bychkov ML, Gasparian ME, Dolgikh DA, Kirpichnikov MP (2014). Combination of TRAIL with bortezomib shifted apoptotic signaling from DR4 to DR5 death receptor by selective internalization and degradation of DR4. Plos One.

[27] Pandit B, Gartel AL (2011). Proteasome inhibitors induce p53-independent apoptosis in human cancer cells. The American journal of pathology.

[28] Zhu Z (2005). Control of ASPP2/(53BP2L) protein levels by proteasomal degradation modulates p53 apoptotic function. J Biol Chem.

[29] Cheng X (2016). Methylisoindigo preferentially kills cancer stem cells by interfering cell metabolism via inhibition of LKB1 and activation of AMPK in PDACs. Mol Oncol.

[30] Cheng X (2014). A TrxR inhibiting gold(I) NHC complex induces apoptosis through ASK1-p38-MAPK signaling in pancreatic cancer cells. Molecular cancer.

[31] Yu J, Zhang L, Hwang PM, Kinzler KW, Vogelstein B (2001). PUMA induces the rapid apoptosis of colorectal cancer cells. Mol Cell.

[32] Hwang PM (2001). Ferredoxin reductase affects p53-dependent, 5-fluorouracil-induced apoptosis in colorectal cancer cells. Nat Med.

[33] Jallepalli PV, Lengauer C, Vogelstein B, Bunz F (2003). The Chk2 tumor suppressor is not required for p53 responses in human cancer cells. J Biol Chem.

[34] Wang W, Kim SH, El-Deiry WS (2006). Small-molecule modulators of p53 family signaling and antitumor effects in p53-deficient human colon tumor xenografts. Proc Natl Acad Sci USA.

[35] Montero J, Dutta C, van Bodegom D, Weinstock D, Letai A (2013). p53 regulates a non-apoptotic death induced by ROS. Cell death and differentiation.

[36] Cheng X (2014). 7,7′-Diazaindirubin–a small molecule inhibitor of casein kinase 2 *in vitro* and in cells. Bioorg Med Chem.

[37] Oliver TG (2011). Caspase-2-mediated cleavage of Mdm2 creates a p53-induced positive feedback loop. Mol Cell.

[38] Yang A, McKeon F (2000). P63 and P73: P53 mimics, menaces and more. Nature reviews. Molecular cell biology.

[39] Richardson PG (2003). A phase 2 study of bortezomib in relapsed, refractory myeloma. N Engl J Med.

[40] Vaziri SA (2009). Inhibition of proteasome activity by bortezomib in renal cancer cells is p53 dependent and VHL independent. Anticancer Res.

[41] Han S (1999). Infrequent somatic mutations of the p73 gene in various human cancers. European journal of surgical oncology: the journal of the European Society of Surgical Oncology and the British Association of Surgical Oncology.

[42] Gong JG (1999). The tyrosine kinase c-Abl regulates p73 in apoptotic response to cisplatin-induced DNA damage. Nature.

[43] Vayssade M (2005). P73 functionally replaces p53 in Adriamycin-treated, p53-deficient breast cancer cells. Int J Cancer.

[44] Irwin MS (2003). Chemosensitivity linked to p73 function. Cancer Cell.

[45] Su X, Chakravarti D, Flores ER (2013). p63 steps into the limelight: crucial roles in the suppression of tumorigenesis and metastasis. Nat Rev Cancer.

[46] Collavin L, Lunardi A, Del Sal G (2010). p53-family proteins and their regulators: hubs and spokes in tumor suppression. Cell death and differentiation.

[47] Kurita T, Cunha GR, Robboy SJ, Mills AA, Medina RT (2005). Differential expression of p63 isoforms in female reproductive organs. Mech Dev.

[48] Dulloo I (2015). Hypoxia-inducible TAp73 supports tumorigenesis by regulating the angiogenic transcriptome. Nat Cell Biol.

[49] Cheng X (2015). Identification of 2-[4-[(4-Methoxyphenyl)methoxy]-phenyl]acetonitrile and Derivatives as Potent Oct3/4 Inducers. J Med Chem.

[50] Cheng X (2015). Ethyl 2-((4-Chlorophenyl)amino)thiazole-4-carboxylate and Derivatives Are Potent Inducers of Oct3/4. J Med Chem.

